# Contemporary endovascular management of common femoral artery atherosclerotic disease

**DOI:** 10.1093/bjs/znab150

**Published:** 2021-05-13

**Authors:** A Saratzis, K Stavroulakis

**Affiliations:** 1 Department of Cardiovascular Sciences, National Institute for Health Research Biomedical Research Centre, University of Leicester, Leicester, UK; 2 Department of Vascular Surgery, Ludwig-Maximilians-University Hospital, Munich, Germany

**Key clinical questions**

This article addresses three modern endovascular modalities that may help the clinician deal with the following particular characteristics of common femoral artery disease, allowing minimally invasive treatment of such lesions:

- Severe calcium deposits in the atherosclerotic plaque (impact on durability of any endovascular treatment)
- Disease of the superficial femoral artery and/or profunda femoral artery with preservation of flow in both vessels after treatment
- Movement of the external iliac and femoral artery when the patient mobilizes/walks (potential for stent fractures).

## Background

Peripheral artery disease (PAD) is the commonest cause of limb amputation^1^^,^^2^. Common femoral artery (CFA) disease is a very common feature of symptomatic PAD. CFA endarterectomy (CFAE) has been the principal mode of treatment for CFA disease over the years. The most common complications of CFAE include major cardiovascular events, given the high-risk profile of patients with PAD, and local complications such as wound infection(s), seroma or lymphocele formation, owing to the location of the procedure and patients’ co-morbidities (diabetes, obesity, frailty)^3^. CFAE may be associated with a combined short-term morbidity and mortality rate as high as 15 per cent, prompting many to think of alternative less invasive treatments^3^. Endovascular or hybrid procedures have lower rates of perioperative complications in the iliac and femoropopliteal segment^3^. The CFA, however, represents a particular challenge for endovascular reconstruction. CFA disease is usually calcified and the superficial/profunda femoris arteries are often involved; it is important that the patency of these arteries is maintained when treating the CFA.

Endovascular devices developed in recent years might help overcome these CFA-specific challenges^3^. Some non-randomized studies have reported outcomes after endovascular CFA treatment^3^. There is significant heterogeneity in terms of the types of procedure performed, follow-up, and reporting; however, early results are promising. One small RCT^4^ has reported that short-term outcomes are better after CFA endovascular treatment compared with surgery. However, the 117 patients taking part were highly selected and the primary endpoint was not focused on clinical effectiveness. In the current literature, short-term major morbidity and mortality rates seem to be higher with CFAE^3^. Endovascular interventions have favourable rates of short-term complications and acceptable technical success, but unfavourable long-term patency^3^. The latter is the main reason why most specialists might still consider CFAE as the standard treatment. Long-term patency after endovascular CFA treatment may be improved greatly by use of newly developed devices, such as intravascular lithotripsy (IVL), biomimetic self-expanding interwoven stent deployment, and directional atherectomy. This has the potential to shift the treatment paradigm for CFA disease.

## Endovascular common femoral artery treatment

### Stenting with biomimetic self-expanding stents

Stent fractures, coverage of the profunda artery origin, and restenosis owing to intimal hyperplasia are potential pitfalls of stenting the CFA^3^. These may be overcome by using a biomimetic stent (for example, Supera stent, Abbott Medical, Illinois, USA). These stents are flexible and have been designed to be deployed within heavily calcified lesions. They are not covered and, therefore, flow into the profunda femoris artery can be preserved. In the event of disease recurrence, CFAE is still feasible. Stenting with such a device distal to the CFA has been shown to achieve good primary patency and durability^5^. Using such a stent for CFA disease, therefore, has the potential to improve immediate/short-term technical success and potentially longer-term patency. The main necessary steps to avoid immediate complications when using this device in the CFA are accurate sizing and lesion preparation with an adequately sized balloon. The potential loss of femoral access for future percutaneous interventions and the risk of compromising (‘jailing’) the profunda femoris artery ostium are the main drawbacks. An ongoing randomized trial^6^ is comparing the performance of the Supera stent with CFAE. Its applicability in a real-world setting remains questionable given that patients with tissue loss will not be recruited into the trial.

### Intravascular lithotripsy

Patency of the profunda femoris artery is paramount in terms of limb salvage in PAD, so a leave-nothing-behind approach might be preferred over CFA stenting. IVL (Shockwave Medical, California, USA) uses pulsatile sonic waves to treat luminal and medial calcium. Lesions in the CFA are typically very calcified. The use of IVL can improve the compliance of the calcified vessel without the need for aggressive vessel preparation, leading to luminal gain with a lower risk of dissection, perforation or embolization. Although IVL is an intuitive system for treatment of severe calcification, oversizing of the IVL catheter (1 : 1.1) plays an important role in procedural success. Of note, IVL catheters are currently available in diameters up to 7 mm. Thus, sufficient oversizing might not be possible for larger CFAs. After application of IVL and improvement in vessel compliance (the calcium has been fractured), the use of an oversized plain balloon is necessary for luminal gain. A restenotic treatment with either a drug-coated balloon (DCB) or a stent can then be considered, depending on the angiographic result. Early observational results have shown that IVL is a safe approach and has favourable early patency^7^. Unpublished results from Mr Konstantinos Stavroulakis an ongoing randomized trial^8^ showed that the rate of stent placement was 4.6 per cent in the IVL group *versus* 18.3 per cent in the plain angioplasty group (*P* < 0.001) in femoropopliteal disease. Overall, 66 per cent of patients in the IVL group achieved the goal of an angiographic diameter stenosis of 30 per cent or less, compared with 50 per cent in the plain angioplasty group (*P* = 0.02). Disadvantages of IVL in the CFA relate to the high immediate cost of the catheters and lack of unproven long-term results.

### Atherectomy

**Fig. 1:**
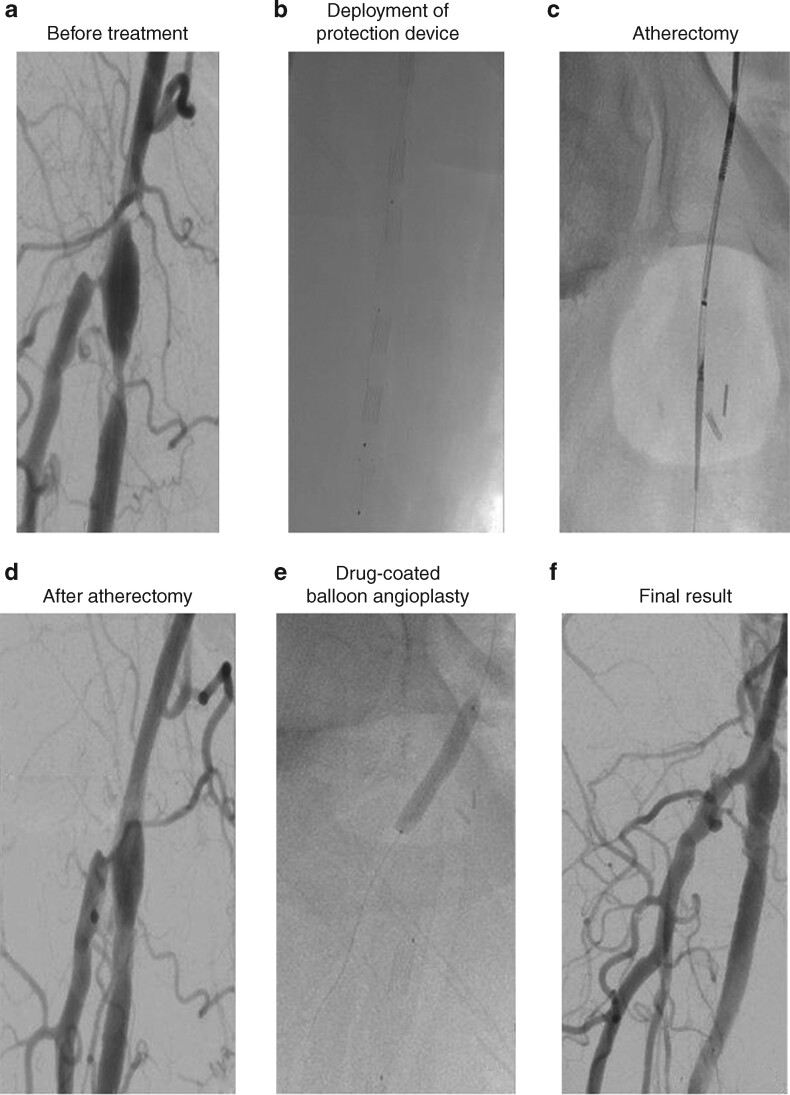
Directional atherectomy with antirestenotic therapy for a femoral lesion proximal to a femoropopliteal bypass anastomosis. a Before treatment. b Deployment of protection device. c Atherectomy. d After atherectomy. e Drug-coated balloon angioplasty. f Final result.

Debulking modalities in combination with drug-coated/eluting therapies are increasingly being used in the CFA. Currently available atherectomy catheters can be used to treat arteries up to 7 mm in diameter; the benefit is that atherectomy does not necessitate subsequent stenting. At the same time, the risk of peripheral emboli does necessitate use of intravascular filters to prevent distal occlusions. Furthermore, some CFAs are larger than 7 mm in diameter. In a retrospective study^9^, the 12-month primary patency rate after vessel preparation with directional atherectomy (to debulk the lesion) before using a DCB was higher than with use of a DCB alone (88 *versus* 68 per cent). However, the difference was not statistically significant. An ongoing RCT^10^ is comparing the performance of directional atherectomy with DCB over CFAE. The inclusion of patients with claudication, however, might limit the reproducibility of the results. The use of a debulking catheter can be particularly helpful in patients with restenosis after CFAE or bypass-anastomosis stenosis, where re-exploring the groin might pose difficulties (*Fig. 1*).

## Conclusion and future developments

Recent advances in endovascular technology allow the treatment of a variety of CFA atherosclerotic lesions, avoiding some of the potential complications of open surgery. Three advances in endovascular technology may help clinicians with minimally invasive treatment of the CFA. Results from ongoing RCTs are awaited to assess the clinical efficacy and effectiveness of these strategies.

*Disclosure*. The authors declare no conflict of interest.
